# Approach to Radical Hysterectomy for Cervical Cancer in Pregnancy: Surgical Pathway and Ethical Considerations

**DOI:** 10.3390/jcm11247352

**Published:** 2022-12-10

**Authors:** Rocco Guerrisi, Sarah Louise Smyth, Lamiese Ismail, Amanda Horne, Federico Ferrari, Hooman Soleymani majd

**Affiliations:** 1 Department of Obstetrics and Gynecology, ‘Filippo Del Ponte’ Hospital, University of Insubria, 21100 Varese, Italy; 2 Department of Obstetrics and Gynaecology, John Radcliffe Hospital, Oxford University Hospital, NHS Foundation Trust, Oxford OX3 9DU, UK; 3 Department of Clinical Oncology, Churchill Hospital, Oxford University Hospitals, NHS Foundation Trust, Oxford OX3 7LE, UK; 4 Department of Obstetrics and Gynecology, “Spedali Civili” Hospital, University of Brescia, 25121 Brescia, Italy; 5 Nuffield Department of Women’s Reproductive Health, Medical Sciences Division, Oxford University, Oxford OX3 9DU, UK; 6 Department of Gynaecological Oncology, Churchill Hospital, Oxford University Hospitals, NHS Foundation Trust, Oxford OX3 7LE, UK

**Keywords:** cervical cancer, pregnancy, radical hysterectomy, case report

## Abstract

Introduction. Cervical cancer is currently the fourth most common cancer in women and in the poorest countries this neoplasia still represents a widespread and potentially lethal disease. We present a rare case of cervical cancer in pregnancy, analyzing the historical changes behind the procedure of radical hysterectomy for cervical cancer and discussing variations in surgical techniques and anatomical definitions that have since been proposed. Results. We present the case of a 33-year-old patient who attended with vaginal bleeding in the second trimester of pregnancy. Examination revealed an abnormal looking cervix, with investigations concluding stage IIb squamous cell carcinoma. Following extensive discussion regarding management options, the patient went on to have a peripartum foetocidal type III nerve sparing radical Wertheim hysterectomy at 18 weeks gestation with conservation and transposition of the ovaries above the level of the pelvic brim. The patient recovered well without significant morbidity and received further input from fertility and psychological medical teams in addition to adjuvant treatment within the department of clinical oncology. Discussion. This case represents several elements of great interest and learning. Notably, we highlight this both due to the surgical challenges that a gravid uterus presents in the execution of a radical hysterectomy; and regarding the compassionate care demonstrated by the team - not only in supporting the patient and her partner in a period of profound turmoil in terms of the management of their cancer diagnosis and unborn child, but also regarding the uncertainty in consideration of the oncological and fertility related outcomes. Conclusion. This manuscript adds to the growing literature on the appropriate use of radical surgery for cervical cancer, more specifically during pregnancy and in consideration of such ethical dilemma, where management guidelines do not exist to aid clinicians further in their provision of treatment.

## 1. Introduction

Cervical cancer, comprising both squamous and glandular differentiation, is currently the fourth most common cancer in women. Although in the most industrialized countries its incidence and related mortality are progressively decreasing thanks to the introduction of screening programs and human papilloma virus (HPV) vaccination; in the poorest countries this neoplasia still represents a widespread and potentially lethal disease [1]. Furthermore, its age-specific incidence rate starts to rise after the age of 25 years old, peaking at 30–34 years of age [2]. For this reason, cervical cancer also represents the most frequent gynecological tumor occurring in pregnancy; with the aggravating circumstance that often the onset of symptoms is confused with other pregnancy-related symptoms, such as post-coital bleeding or abnormal unspecified vaginal bleeding during pregnancy or the postpartum period [3]. Nevertheless, the “relative” rarity of the disease and the lack of randomized controlled studies have led to the absence of established treatment guidelines. The management of cervical cancer in pregnancy mainly follows the principles for that of the non-pregnant patient, enhanced only by limited clinical case reports, expert opinions, and through the sharing of therapeutic strategies in the context of multidisciplinary teams (MDT) [4].

Using a case example, we present this surgical approach to peripartum foetocidal type III nerve sparing radical Wertheim hysterectomy in advancing gestation, whilst also taking into consideration the ethical dilemmas of management of cervical cancer in pregnancy.

This presentation was additionally compounded in view of delays secondary to the COVID-19 pandemic and acknowledges further the holistic aspects and therapeutic implications affecting both the patient and clinical team.

## 2. Case Report

### 2.1. Patient Information

We present the case of a 33-year-old woman. The patient has no previous medical or surgical history of note. She is a non-smoker with a normal body mass index of 21.7. She is primiparous at 16 weeks pregnant and has had an uneventful pregnancy to date, with a normal nuchal ultrasound scan. The patient presented with painless vaginal spotting, reporting a bulge within the posterior vaginal wall on self-examination. 

### 2.2. Clinical Findings

Examination revealed concerns regarding the appearance of her cervix, which was raw with the os not identified and significant contact bleeding. A referral was made to colposcopy. On further questioning, whilst the patient’s smear test was overdue and delayed due to the COVID-19 pandemic, the most recent test was normal four years prior.

### 2.3. Diagnostic Assessment

Colposcopic examination revealed evidence of at least stage Ib3 exophytic cervical cancer. A biopsy of the tissue histologically reported a highly atypical squamous epithelium with full thickness dysplasia and multiple mitoses (>50 per 10 high power fragments). Several fragments contained islands of atypical epithelial cells in fibrous stroma, which were highly suspicious of invasion. The conclusion was of invasive HPV associated squamous cell carcinoma.

**Figure 1:**
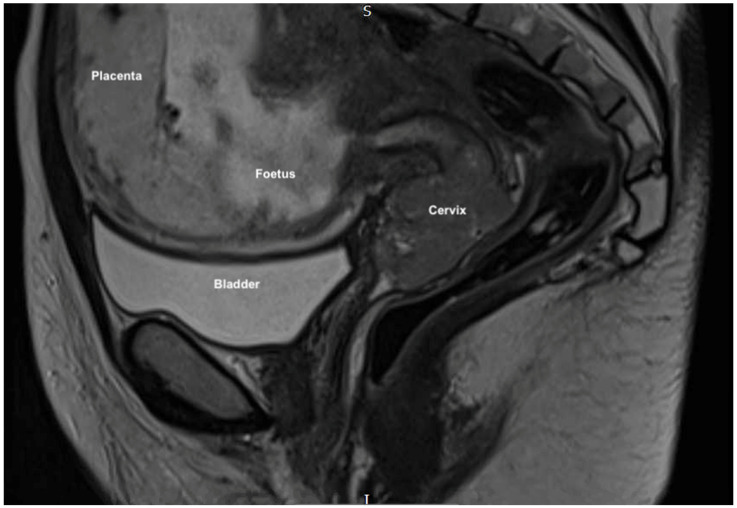
MRI sagittal scan showing the gravid uterus, anteverted and anteflexed, with a single foetus in cephalic presentation with cervical mass seen distally.

**Figure 2:**
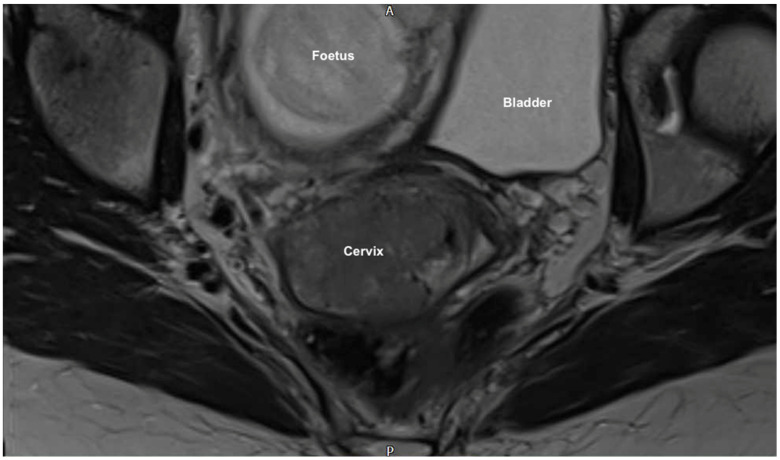
MRI coronal scan showing the large cervical mass (38 × 53 × 25 mm) centered on the ectocervix with probable parametrial extension. It fills the vaginal fornix and extends into the cervical canal also.

Magnetic resonance imaging (MRI) pelvis reported an anteverted anteflexed gravid uterus with a single foetus in cephalic presentation. A large mass was seen centered on the ectocervix filling the vaginal fornix and extending into the cervical canal measuring 38 × 53 × 25 mm. (Figure 1 and Figure 2) There was also felt to be possible parametrial extension at the superior right aspect of the mass. There was no evidence of lymphadenopathy or metastatic disease, which was further confirmed on computed tomography (CT) scan of the chest, abdomen and pelvis. MDT discussion confirmed radiological stage IIb disease and management proposals were considered.

### 2.4. Timeline of Events

The patient and her partner subsequently met with gynecological oncology surgery, high risk obstetric and clinical oncology specialists alongside cancer nurse specialist (CNS) and senior midwifery support. They were informed that standard treatment outside of pregnancy would be with primary chemoradiotherapy and brachytherapy, which would require termination of the pregnancy in consideration of evidence that spontaneous delivery is reported to have a negative prognostic impact in patients with cervical cancer in pregnancy [5].

Three main treatment options were discussed in depth:

- Continuation of the pregnancy with neoadjuvant chemotherapy (which evidence suggests would not cause significant harm to the foetus with a response rate of approximately 70%) [6] until three weeks prior to delivery via caesarean hysterectomy at approximately 32–34 weeks, followed by combined chemoradiotherapy and brachytherapy;
- Termination of the pregnancy with direct hysterotomy or with ultrasound guided foetal intracardiac potassium chloride injection and subsequent attempted vaginal delivery, both options followed by combined chemoradiotherapy and brachytherapy;
- Termination of the pregnancy (due to the risks associated with vaginal delivery) with midline laparotomy, type III radical Wertheim hysterectomy, bilateral salpingectomy, oophorectomy (pending surgical findings) and bilateral pelvic and para-aortic lymphadenectomy, likely followed by combined chemoradiotherapy and brachytherapy.

Further consideration was made to potential conservation and transposition of the ovaries, with fertility preservation specialist input sought and options discussed including ovarian tissue storage with possible reimplantation, donor eggs or adoption. The patient was also advised that, were the pregnancy to continue, there remained to be substantial risks associated with preterm delivery alongside risk of significant bleeding, side effects of chemotherapy and risks of even further prematurity and miscarriage (with associated sequalae of potential disability or death) were the patient to present as an obstetric emergency. In fact, to this end, the patient subsequently presented and was admitted for observation following a significant antepartum hemorrhage, further highlighting the risk of significant bleeding. The patient and her partner also raised concerns regarding potential risk to the patient’s survival should the pregnancy continue owing to treatment delay. 

The patient and her partner were provided with information regarding support services including ‘Maggie’s Centre’, ‘Jo’s Trust’, and the CNS and midwifery bereavement team. It was recognized that there was minimal local social support for the couple. Following very difficult decision making, with the input of their relatives, the couple felt that they wished to prioritize the patient’s health over that of the foetus, both regarding risks of the cancer itself but also those associated with continuation of the pregnancy. It was proposed that this was significantly influenced by her admission for antepartum hemorrhage. Following further MDT discussion, a plan was made for foetocide and surgical treatment, with the full support and understanding of the medical team. The patient subsequently declined pre-operative foetocidal ultrasound guided intracardiac potassium chloride injection. 

### 2.5. Therapeutic Intervention

At eighteen weeks and six days gestation the patient underwent midline laparotomy, peripartum foetocidal radical type III nerve sparing Wertheim’s hysterectomy, upper vaginectomy, bilateral salpingectomy, bilateral pelvic and para-aortic lymphadenectomy and conservation and transposition of the ovaries above the level of the pelvic brim under general anaesthetic. 

There was an intense atmosphere present within the operating theatre—performing a surgery (whose purpose was well known) with direct effect on a healthy foetus of 18 weeks gestation, was one of the most complex aspects to manage for all team members. Obviously, to all of this must be added the technical complexity of the surgery and the emotional responsibility towards a most unusual case. The supportive environment that the team created was fundamental and, even more, the support that they gave each other.

**Figure 3:**
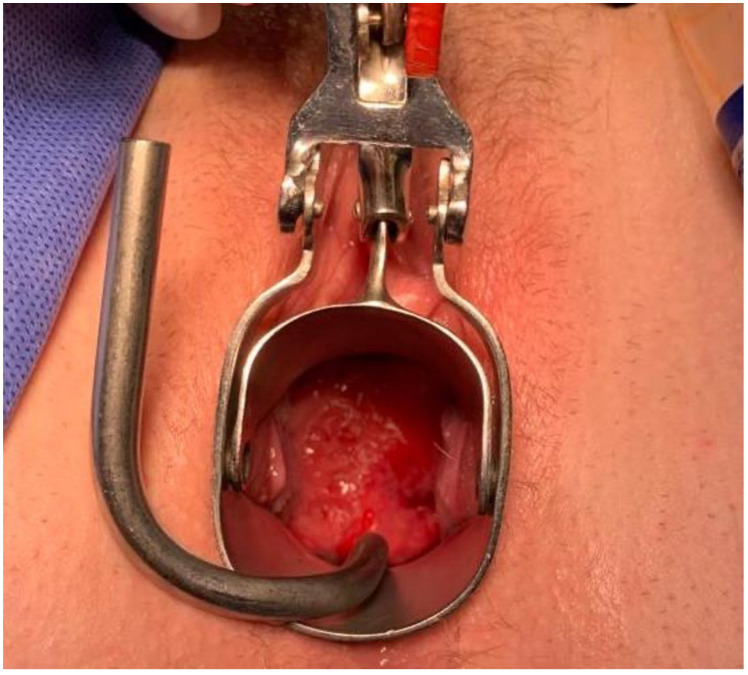
Vaginal assessment of the cervix, which appears to be completely replaced by the 5 cm exophytic tumor.

**Figure 4:**
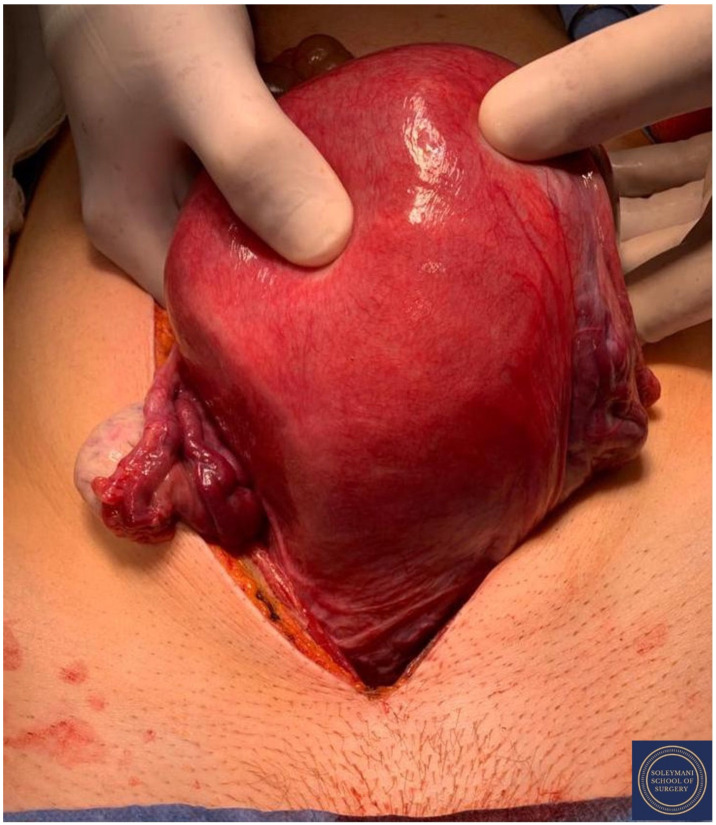
The eighteen-week gravid uterus with foetus inside.

**Figure 5:**
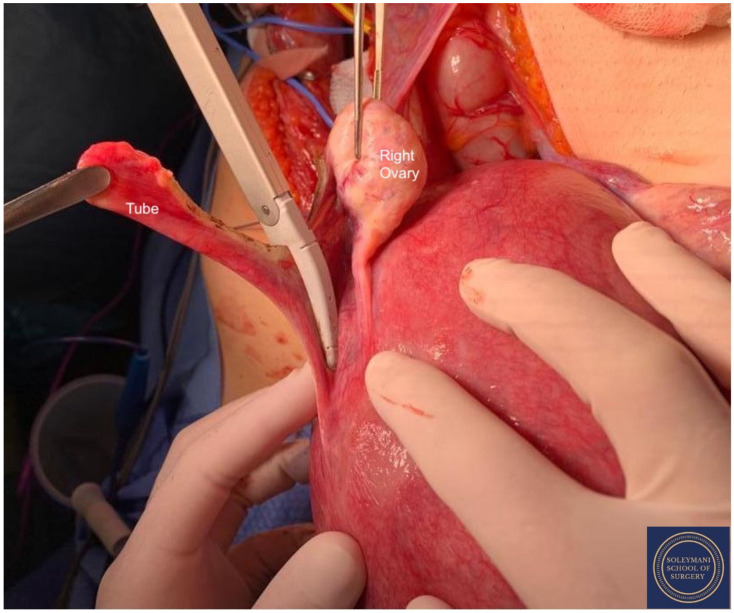
The ovaries appeared bilaterally normal allowing for their preservation with bilateral salpingectomy and division of the tuboovarian ligaments.

**Figure 6:**
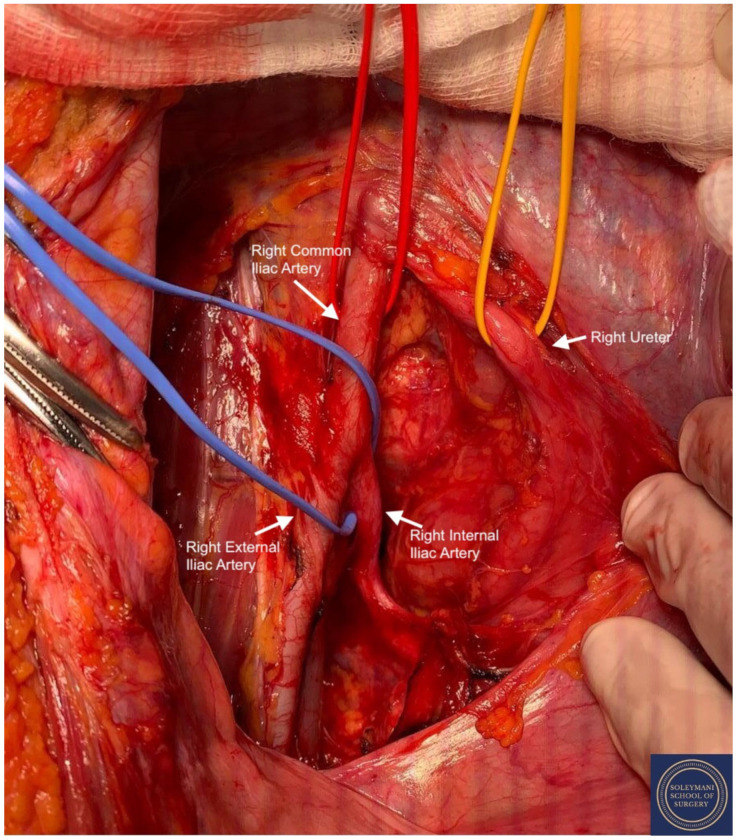
Exposure of the right pararectal space with isolation of the following structures: Red Sling = right common iliac artery, Yellow Sling = right ureter, Blue Sling = right internal iliac artery.

**Figure 7:**
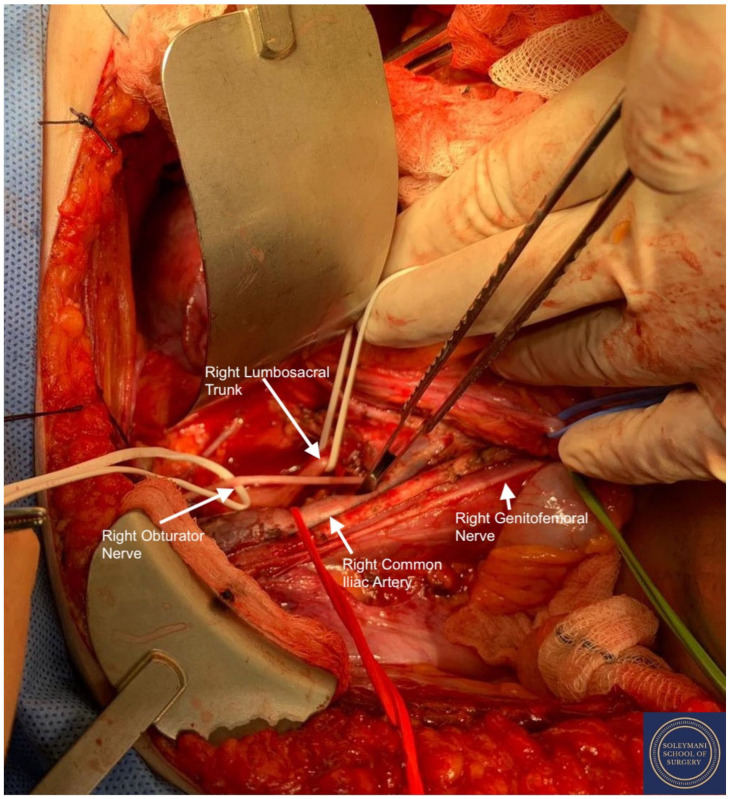
Full exposure of the right lumbosacral fossa with isolation of the following structures: Red Sling = right common iliac artery, Green Sling = right genitofemoral nerve, Upper White Sling = right lumbosacral trunk. L4 L5 S1 S2 roots and sciatic nerve, Lower White Sling = right obturator nerve.

**Figure 8:**
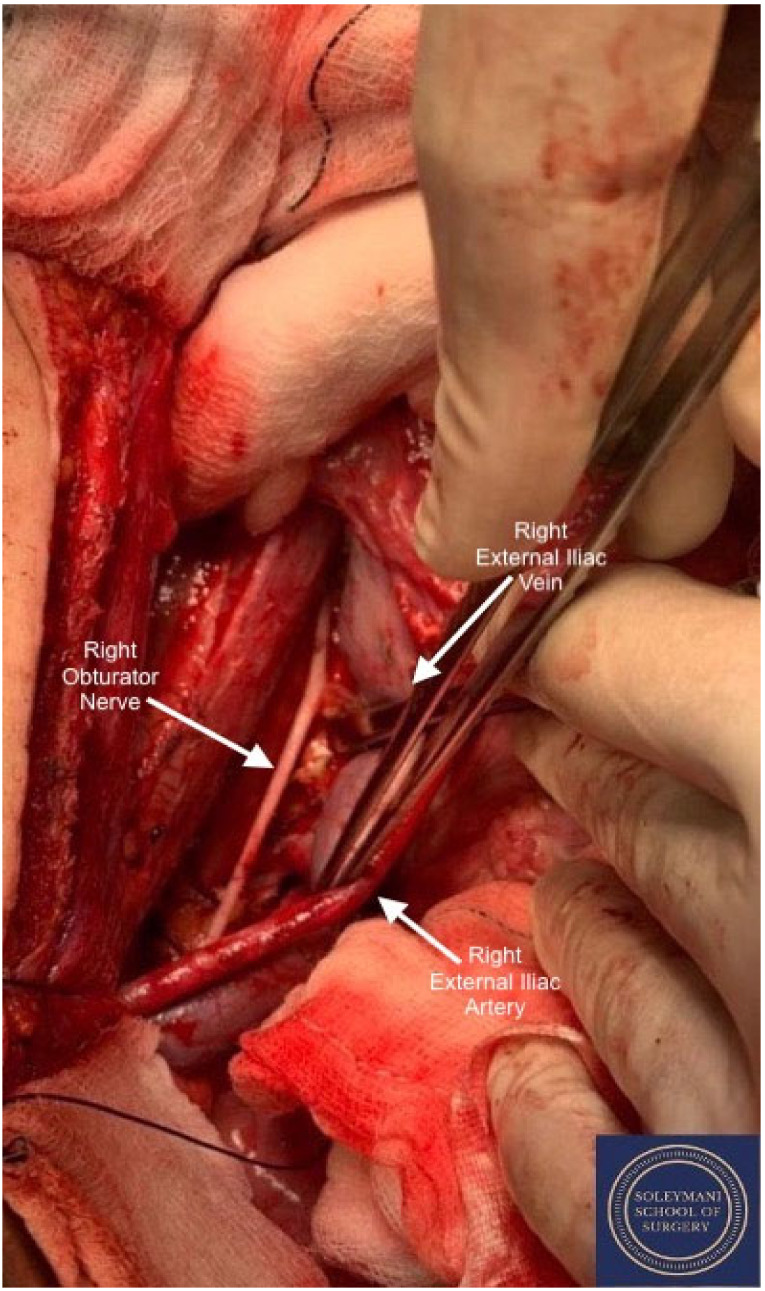
Detail of the right obturator fossa.

**Figure 9:**
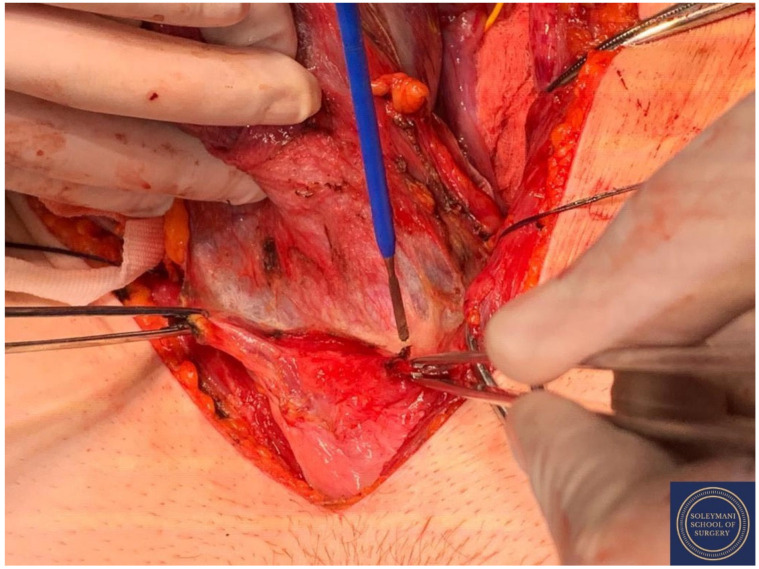
Incision of the peritoneum of the vescicouterine pouch with complete mobilization of the bladder away from the anterior cervix and the proximal vagina.

**Figure 10:**
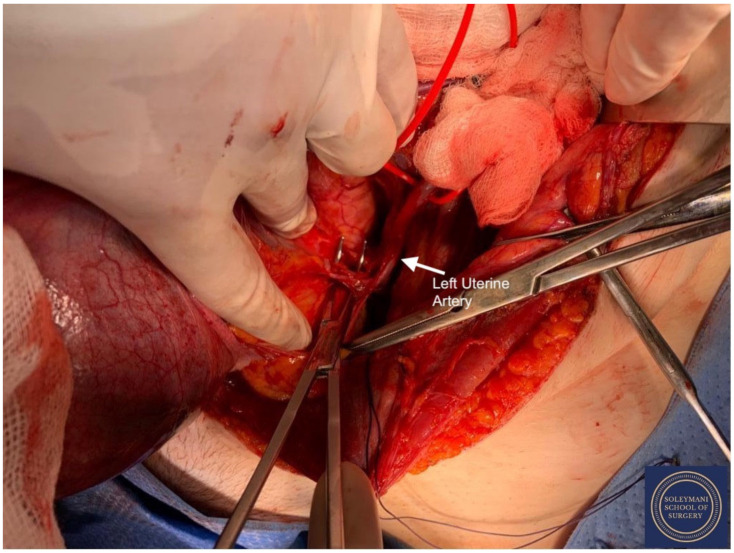
Exposure of the left uterine artery at origin from the internal iliac artery prior to division.

**Figure 11:**
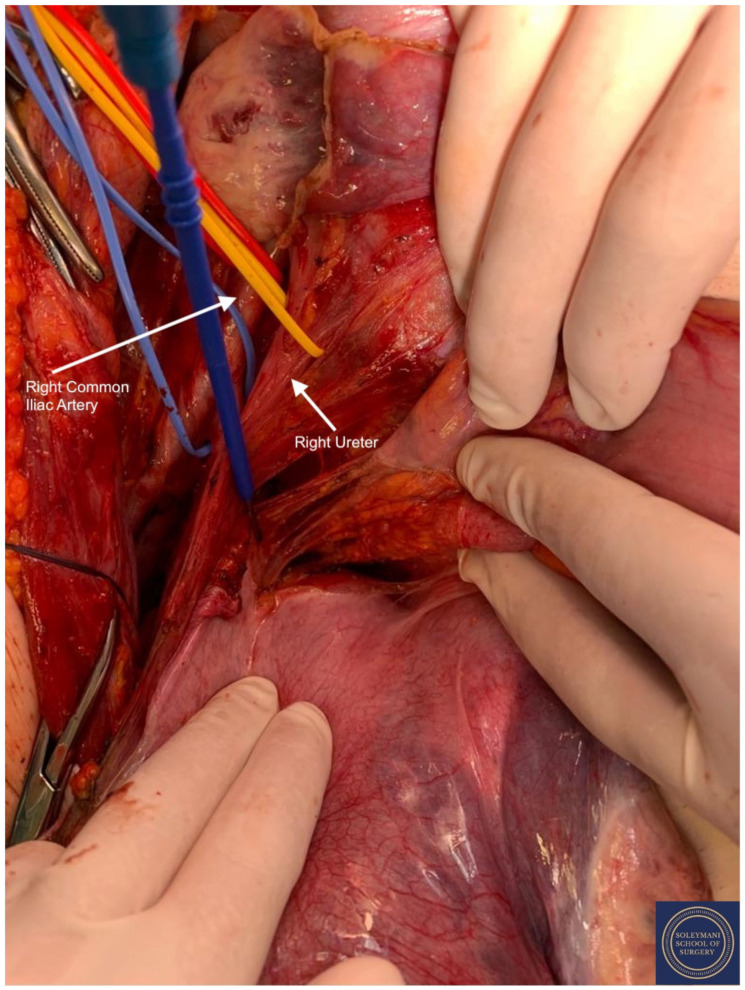
Separation of the mesorectum from the uterosacral ligaments to the level of the inferior hypogastric plexus. Isolation of the following structures: Red Sling = right common iliac artery, Yellow Sling = right ureter.

**Figure 12:**
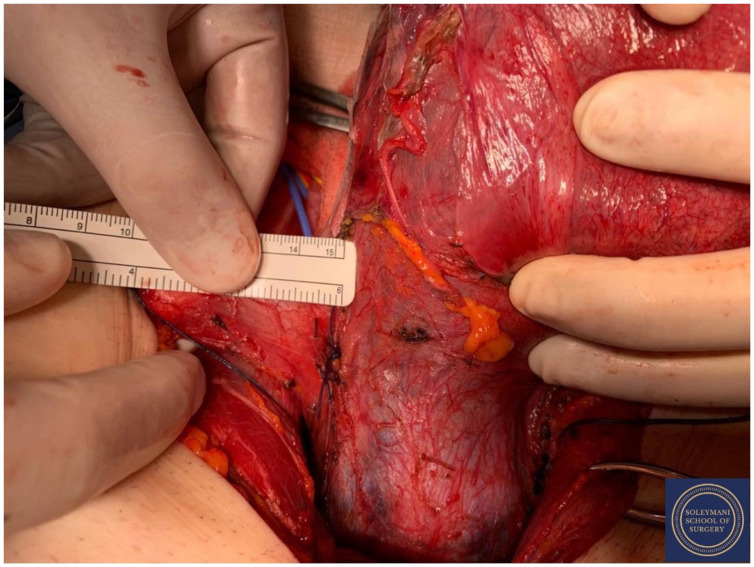
Complete dissection of the right parametrium.

**Figure 13:**
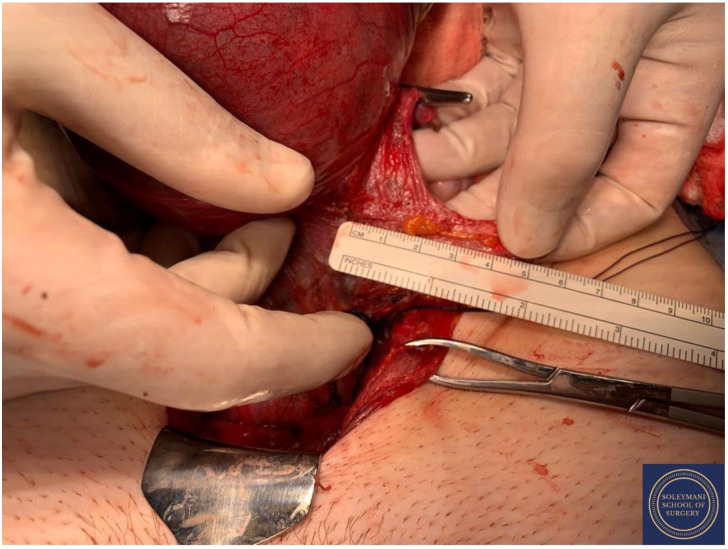
Complete dissection of the left parametrium.

**Figure 14:**
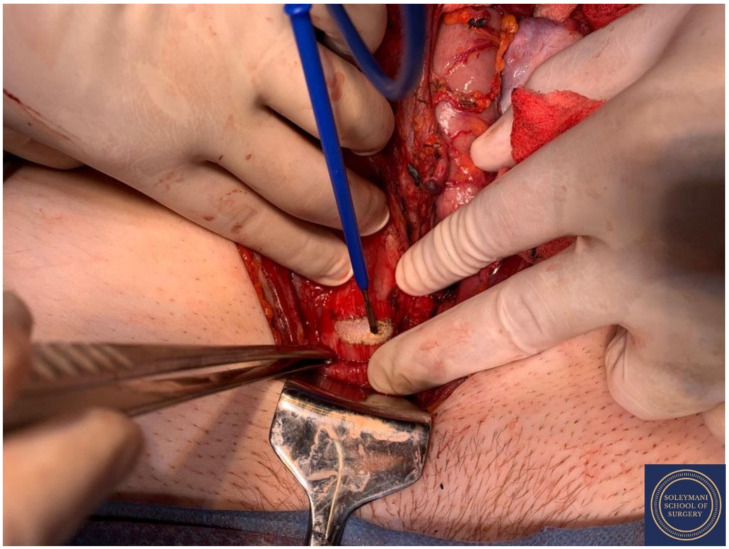
The colpotomy was approached anteriorly using a circumferential incision.

**Figure 15:**
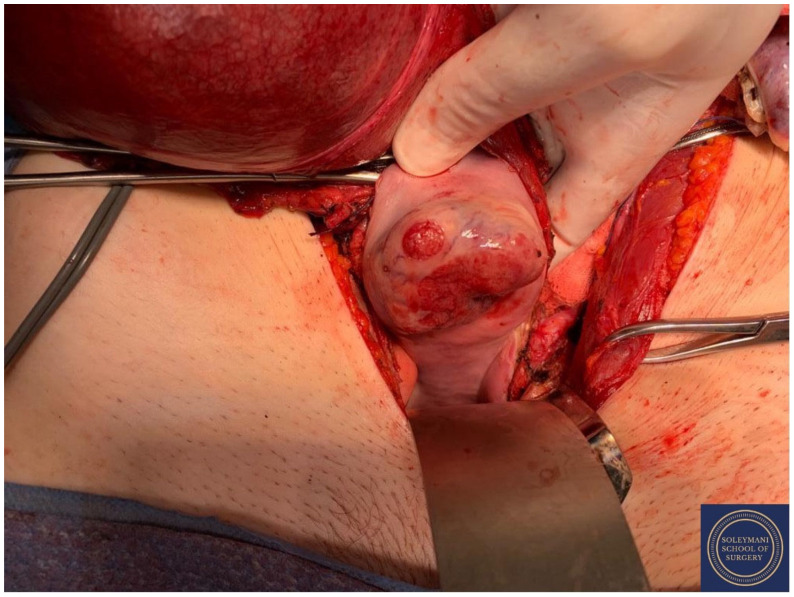
Upper vaginectomy ensuring an appropriate excision margin.

**Figure 16:**
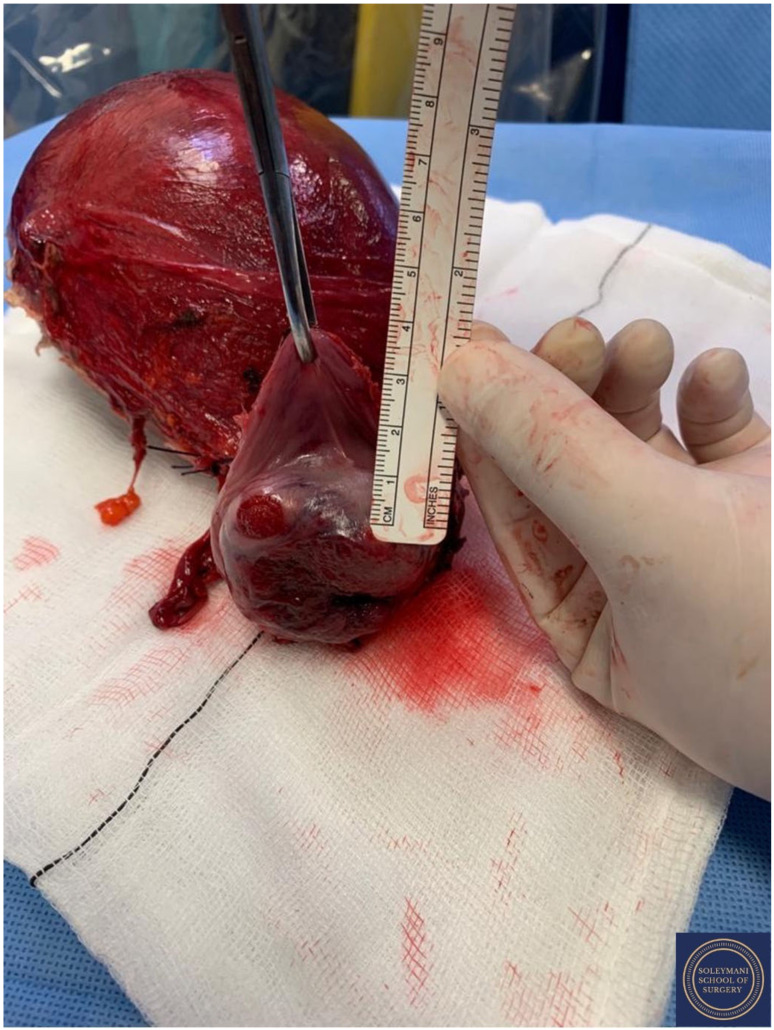
Appearance of the vaginal cuff on the final specimen.

**Figure 17:**
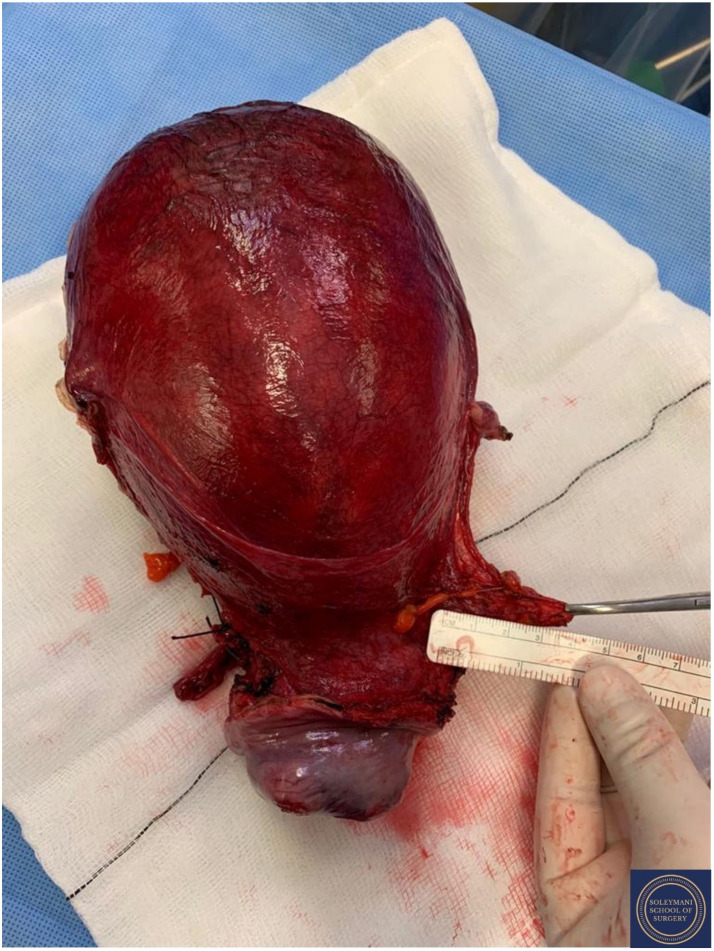
Appearance of the left parametrium and the corpus uteri prior to performance of hysterotomy and delivery of the foetus.

**Figure 18:**
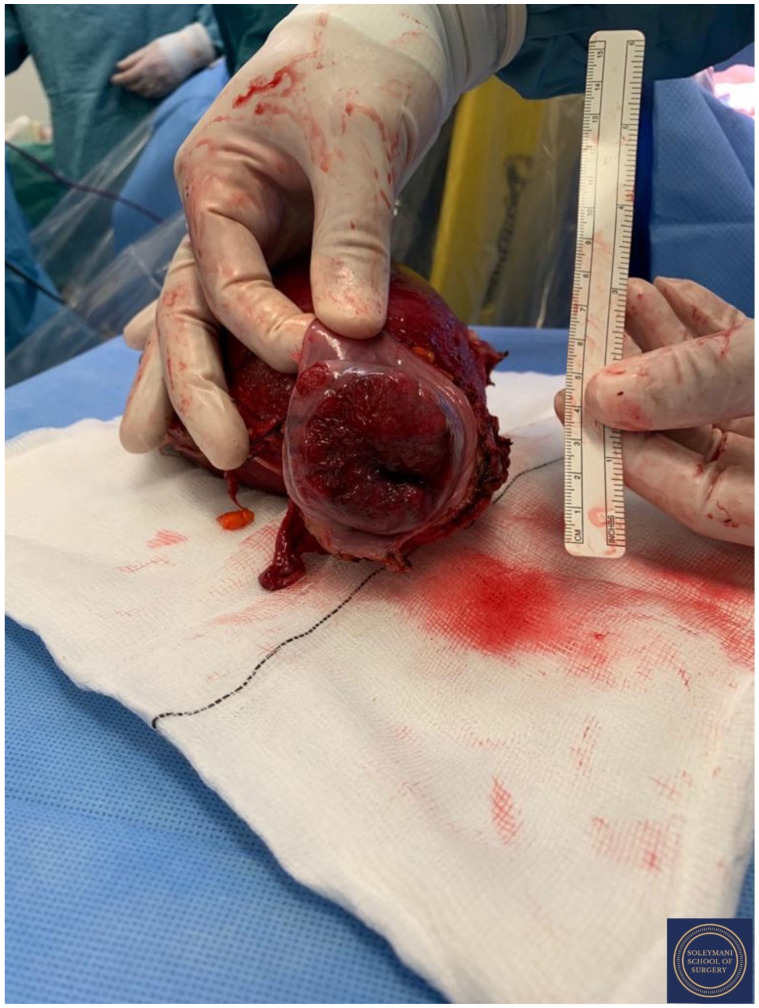
Appearance of the cervical exophytic mass on the final specimen.

Findings at the time of surgery were of a 5 cm exophytic tumor, which had completely replaced the ectocervix and an eighteen-week gravid uterus. (Figure 3 and Figure 4) We report below the surgical steps that were carried out according to the description of Piver, Rutledge and Smith Class III and total mesometrial resection (TMMR)/extended mesometrial resection (EMMR) [7,8] with the necessary adjustments in relation to the particularity of our case:

- The patient was positioned in modified Lloyd Davis and catheterized. Surgical access was gained via a midline incision extending above the level of the umbilicus.
- Findings were of an exophytic 5 cm tumour, which had completely replaced the ectocervix and an 18-week gestation gravid uterus. Pelvic and abdominal structures were otherwise normal in appearance.
- The pelvic sidewall and mid-abdominal retroperitoneum were opened by incising the peritoneum at the psoas muscles, paracolic gutters, and along the mesenteric root and Todlt line to the level of L1, revealing the main pelvic avascular spaces (Table 1) [9,10] with full exposure of the inferior vena cava and common iliac vasculature.
- Exposure of the infundibulo-pelvic ligaments and ureters were achieved with mobilization of the cecum, duodenum, and descending and sigmoid colon to the level of the common iliac vessel bifurcation with identification of the superior hypogastric plexus. The ureters, common iliac and internal and external iliac vasculatures were slung.
- The round ligaments were transected and the anterior and posterior leaves of the broad ligaments were incised. The ovaries were normal in appearance, allowing conservation with bilateral salpingectomy and division of the tuboovarian ligaments. They were secured above the level of the pelvic brim (Figure 5).
- Anteriorly, the paravesical spaces were developed with the umbilical arteries adhering medially to the bladder, exposing the complete anterior side of the urogenital mesentery. The umbilical artery together with the superior bladder mesentery were both separated from the anterior mesometrium.
- The pararectal spaces were developed with exposure and preservation of the hypogastric nerves adhering medially to the mesorectum up to the level of the inferior hypogastric plexus and vein aergentis. The external iliac and obturator lymph nodes of the anterior pelvic compartments (Figure 6) were removed by completely stripping the external iliac artery and vein and removing the paravisceral pelvic fat pads, obtaining exposure of the obturator nerve, obturator artery and vein, the arcus tendineus, and proximal sciatic nerve (Figure 7 and Figure 8).
- Further ureterolysis was performed to skeletonize the ureters distally to the level of the bladder insertion. The peritoneum of the vescicouterine pouch was incised and the bladder was fully mobilized and separated from the anterior cervix and the proximal vagina with division of the vesicovaginal pillars (Figure 9).
- The uterine arteries were identified at origin and secured (Figure 10). The peritoneum of the pouch of Douglas was incised and the anterior mesorectum was separated from the posterior vaginal wall with division of Dennonveiliers fascia down to the mid-vagina. Laterally, the mesorectum was separated from the uterosacral ligaments to the level of the inferior hypogastric plexus, which was subsequently mobilized from these ligaments from both proximal and lateral aspects (Figure 11). Immediately above the superior margin of the inferior hypogastric plexus, the rectouterine ligaments and uterosacral ligaments were subsequently transected in a stepwise fashion.
- Anteriorly, the mesometrium was mobilized from its origin at the site of the already transected uterine arteries and veins towards the uterus and beyond the superior surface of the ureter. The vesicovaginal venous plexus together with the dense sub-peritoneal connective tissue above the prevesical segment of the ureter was ligated and divided. Thus completed the formation of the anterior, posterior and lateral parametrium (to a depth of 5 cm) (Figure 12 and Figure 13), paracolpium and vaginal cuff.
- Using a circumferential incision, colpotomy was approached anteriorly (Figure 14) with retrograde Hudson hysterectomy and upper vaginectomy (Figure 15), ensuring a 3 cm excision margin (Figure 16). The vaginal vault was sutured in routine fashion.
- Pelvic lymph node dissection was continued in the posterior compartment by removing all lymph nodes and fatty tissue around the internal and common iliac vessels, exposing the proximal pelvic obturator nerves and the lumbar rami of the sacral plexus. Para-aortic lymph node dissection was subsequently completed up to the level of the renal veins.
- Following confirmation of haemostasis with washout and irrigation, a size 16 Robinson’s drain was inserted. The laparotomy was closed in layers with loop PDS, interrupted single Maxon and skin staples with a total estimated blood loss of 100 mL. A hysterotomy was performed following hysterectomy with the delivery of an infant weighing 203g. Appearances were in keeping with gestation. [11] All specimens were sent to histopathology urgently (Figure 17 and Figure 18).

**Table 1:** The pelvic avascular spaces [9,10].

Medial Paravescical Space	The separation of the wider Paravescical Space into these two spaces is given by the passage of the obliterated umbilical artery.	The boundaries of these spaces considered together are: Ventrally—superior pubic ramus, arcuate line of the os ilium; Dorsally—cardinal ligament including parametrium (over the ureter) and paracervix (below the ureter), uterine artery/vein; Medially—caudal portion of vesico-uterine ligament, bladder; Laterally—obturator internus fascia/muscle, external iliac artery/vein.
Lateral Paravescical Space		
Medial Pararectal Space (Okabayashi Space)	The separation of the wider Pararectal Space into these two spaces is given by the passage of the ureter.	Its boundaries are: Ventrally—cardinal ligament; Dorsally—presacral fascia, sacrum; Laterally—ureter, mesoureter; Medially—uterosacral ligaments.
Lateral Pararectal Space (Latzko Space)		Its boundaries are: Ventrally—cardinal ligament; Dorsally—presacral fascia, sacrum; Laterally—internal iliac artery; Medially—ureter, mesoureter.
Yabuki Space	Also known as the fourth space.	There are still controversies around its exact location but it should be found between the the cranial portion of the vesicouterine ligament and the ureter.
Retropubic Space	Also known as Retzius Space	Its boundaries are: Ventrally—pubic symphysis; Dorsally—parietal peritoneum, bladder; Cranially—transversals fascia; Caudally—urethra, adjacent pubocervical fascia and bladder neck; Laterally—the arcus tendinous fasciae pelvis.
Vescicovaginal Space	Also known as anterior cul-de-sac	Its boundaries are: Ventrally—bladder; Dorsally—pubocervical fascia, cervix/vagina; Laterally—cranial portion of vesicouterine ligament; Cranially—peritoneal reflection between the dome of the bladder and the lower uterine segment; Caudally—junction of the proximal and middle thirds of the urethra.
Rectovaginal Space	Also known as posterior cul-de-sac	Its boundaries are: Ventrally—posterior vaginal wall; Dorsally—anterior rectal wall; Laterally—uterosacral ligaments (cranial), rectovaginal ligament (caudal); Cranially—peritoneal reflections of the pouch of Douglas; Caudally—levator ani muscle.
**Retrorectal Space**	Also known as presacral space	Its boundaries are: Ventrally—mesorectal fascia/rectum; Dorsally—longitudinal anterior vertebral ligament, sacral promontory; Laterally—right (right common iliac artery/right ureter), left (left common iliac vein/left ureter), hypogastric fascia, which is formed by the medial fibers of the uterosacral ligaments; Cranially—peritoneal reflection of the rectosigmoid colon; Caudally—levator ani muscle.

### 2.6. Follow Up and Outcomes

Post-operatively the patient was initiated on a four-week course of low molecular weight heparin. Trial without catheter failed on day two but was subsequently successful on day five and the patient was treated with oral antibiotics for a suspected urinary tract infection. The Robinson’s drain was removed on day three and a mild ileus was managed conservatively. The patient was reviewed regularly by the bereavement midwifery team regarding birth choices including seeing the baby, memory photos, involvement in funeral plans and birth registration. Bereavement counselling was also arranged. The CNS team continued to review in respect of how the patient and her partner were supporting each other alongside emotional management of the loss of their baby. It became apparent that their main focus was that of future aspects of infertility. The patient was discharged on day seven.

Histopathology reported a grade 3 squamous cell carcinoma with the following figures: maximum horizontal dimension was 43 mm; the depth of invasion was 27 mm; deep cervical stromal involvement and lymphovascular space invasion (LVSI) were both present. 26 of 26 pelvic/lumbosacral/aorto-caval lymph nodes were negative; 1 out of 1 right parametrial lymph node was positive; 1 out of 1 left parametrial lymph node was negative (total lymph nodes were 28). All vaginal margins were clear. The right parametrium, which measured 50 × 20 × 5 mm, was involved by the tumour; the left parametrium, which measured 75 × 30 × 11 mm, was disease free. The closest vaginal margin was 7 mm; the vaginal cuff was 20 mm posteriorly and 17 mm anteriorly. The MDT concluded this to be histopathological stage IIb disease with a recommendation for referral to clinical oncology for consideration of adjuvant treatment.

The patient received post-operative follow-up review in the gynecological oncology clinic, where she reported to have recovered well from surgery with no on-going bladder or bowel symptoms, surrogate markers of nerve sparing [12]; and with unremarkable examination findings. She was informed of the MDT recommendation for positron emission tomography (PET) CT scan and onward referral for radical chemoradiotherapy. This demonstrated postsurgical appearances of the pelvis with a mildly fluorodeoxyglucose (FDG) avid focus in the right lobe of the liver suspicious for metastatic disease. A liver MRI remained concerning despite acknowledgment that the lack of definite abnormality was atypical. In the interim, referrals were also made to the fertility team for ovarian stimulation and egg retrieval prior to adjuvant treatment and to psychological medicine specifically regarding support surrounding the loss of fertility. This yielded two viable embryos. Upon further discussion with the oncology team, a liver biopsy was felt to be technically challenging and may not subsequently have provided reassurance even with a negative result. Therefore, on balance it was felt likely that this lesion represented a variation of normal physiology only, with stable appearances on subsequent MRI and PET scans. Following adjuvant treatment, the patient was referred to both the early menopause and lymphoedema clinics. She continues under surveillance.

## 3. Discussion

In consideration of the surgical approach to peripartum foetocidal type III nerve sparing Wertheim hysterectomy in advancing gestation; we reflect on the evolution of various anatomical descriptions and surgical classifications regarding cervical cancer and radical hysterectomy.

“Radical hysterectomy” in the surgical management of cervical cancer was first described by Wertheim in 1912 [13] and, only later, by Okabayashi and Meigs, respectively in 1921 and 1944 [14,15]. The procedures described by these authors continue to represent a gynaecological oncology milestone in the mainstay of our practice, however we recognize their limitations and potential confusion in alternating nomenclatures, whilst referring to identical anatomical structures. In 1974, Piver, Rutledge and Smith presented their classification of radical hysterectomy in five categories, which, whilst still referred to in current terms also hosts limitations in consideration of nerve and fertility sparing techniques, alongside the potential need to alternatively classify treatments, which did not fall into said categories [16].

For these reasons, in 2008, Querleu and Morrow proposed their own classification of radical hysterectomy (later updated in 2017), based only on the lateral extension of the resection. They described four types (A–D), adding, when necessary, subtypes that considered nerve sparing and paracervical lymphadenectomy. Furthermore, the classification itself was potentially applicable to fertility-sparing surgery and could additionally be adapted to open, vaginal, laparoscopic or robotic surgery [17]. Subsequently, in 2011, to remedy the lack of description of three dimensional parametrial resection (in consideration of post-operative morbidity), Cibula further developed this using standard anatomical reference points for the definition of the resection margins both in longitudinal and transverse planes, further promoting the achievement of a universal classification for radical hysterectomy [18].

Parallel to this and dating back to 2003, drawing inspiration from the results achieved in the surgical treatment of rectal cancer by the introduction of the total mesorectal excision (TME), Hockel began to develop a new theoretical model for the realization of radical hysterectomy. Through Hockel’s analysis of uterovaginal development in serial sections of female human embryos and foetuses, he concluded that spatial extension of the Müllerian mesenchyme, its vascularization, and its innervation during early uterovaginal organogenesis determined a tissue territory that can be followed during foetal development and identified in women as a morphogenetic unit. 

From these ontogenetic-anatomic considerations, he introduced the total mesometrial resection (TMMR) and the laterally extended endopelvic resection (LEER). Hockel demonstrated that the radical en-bloc resection of this Müllerian compartment led to local tumor control, preservation of autonomic nerves, and a reduced need for adjuvant radiotherapy. Just after this, for a subset of patients with locally advanced disease, he proposed an operative strategy characterized by the resection of additional tissue at risk for tumor infiltration as compared to TMMR, but less than in LEER, that he defined as the extended mesometrial resection (EMMR) further promoting preservation of bladder function [7,11,19,20,21]. Moreover, in 2022 the first results from the TMMR register study have been published revealing that the outstanding oncologic data described for the TMMR have also been reproducible in this multicentric setting [8].

This case represents several elements of great interest and learning, which in combination present a complex management strategy from multiple aspects. Notably, we highlight this due to the surgical challenges that an 18-week gravid uterus presents in the execution of a radical hysterectomy. Additionally, we acknowledge the compassionate care demonstrated by the team despite a significant emotional burden, not only in supporting the patient and her partner in a period of profound turmoil regarding the management of their cancer diagnosis and unborn child; but also the uncertainty in consideration of the oncological and fertility related outcomes.

The number of cancers diagnosed during pregnancy has been increasing in recent years, and although figures relating to cancer stage, grade, etc. continue to deserve attention even in such a particular context, the main complication of these diagnoses is represented by the pregnancy itself. The occurrence of these two events—cancer and pregnancy—in combination, creates a challenging conflict between maternal care and foetal well being. The optimum outcome is therefore represented by the achievement of similar oncological outcomes as with non-pregnant cases, in consideration of ongoing experimental techniques under circumstances where a paucity of evidence is available on the subject.

Our first consideration is the historical moment and situation in which the patient found herself. In fact, as we continue through the COVID-19 pandemic, a thriving literature has been produced on the delay of cancer diagnoses and consequential delay in the treatment of neoplastic diseases all over the world: compared to pre-pandemic data, a substantial increase in the number of deaths is estimated owing to diagnostic delay [22]. A significant number of major screening programs have been suspended during the pandemic, and although recovery strategies have been proposed, [23] we do not know how many cancer cases could have been diagnosed at an earlier stage. Our patient is well educated, having adhered to pre-pandemic screening programs. Although some data suggests that a deferred smear due to pregnancy is acceptable practice in the UK and that a diagnosis of cervical cancer during pregnancy does not affect the overall survival rate, [24] an earlier diagnosis may have resulted in a host of alternative outcomes from multiple perspectives. 

One of the most important concerns regarding the clinical management of this case was in terms of treatment options. As we previously reported, the treatment of cervical cancer occurring in pregnancy mainly follows the principles as for the state of non-pregnant disease. In holistic consideration of this patient’s best interests, we were clear in the pre-operative radiological diagnosis of stage IIb cervical cancer, which should be treated with primary chemoradiotherapy and brachytherapy according to ESMO/ESGO/ESP guidelines [5].

Despite this, in consideration of her pregnancy, we were acutely aware of the ethical dilemmas faced regarding aspects of management and the challenges associated with maintaining absolute rationality in these circumstances, both from the perspectives of the patient and her partner as well as the clinicians. As we have already reported before, the main options offered to the patient were therefore chemotherapy in order to stabilize and prevent the progression and spread of the tumor, with a subsequent expedition of delivery by caesarean section at 34 weeks due to the increased risk of premature rupture of membranes or myelosuppression, followed by further chemoradiotherapy [25]; foetocide and attempted vaginal delivery or the adoption of a surgical treatment. Moreover, except for the experiences published in 1992 and 2009 by two other groups on partially similar cases, [26,27] this type of approach is certainly a novelty in the treatment of cervical cancer during pregnancy [28]. Considering the likely necessity to defer back to a surgical procedure (to terminate the pregnancy and deliver the foetus), the patient opted for surgical treatment in the full knowledge that this would probably not offer definitive treatment. For the professionals who cared for her, this represented a truly complex event both from a technical perspective, given the uniqueness of the procedure performed on a gravid uterus, as well as from a psychological point of view.

This runs in parallel to the significant challenges associated with the management of a vast plethora of emotions that a cancer diagnosis presents. Whilst a challenging life event in itself, to contemplate this alongside pregnancy in a young and otherwise healthy woman constituted much more difficult circumstances. In view of this rarity, empathy is not often understood, and this intense psychological stress can create widely deleterious effects on the mother and the offspring. Moreover, the effect on the partner, was also acknowledged, potentially threatening the couple’s stability [6,29]. Further still, beyond the emotional burden for the patient and her partner, we must additionally consider the involvement of a third individual who, however, has no capacity for expression at this point. From the perspective of our team, the ethical dilemma of management of the foetus generated huge psychological pressure. This was the couple’s first pregnancy, a desired pregnancy, and approaching the choice between the patient’s own health and continuing the pregnancy despite the associated risks for the mother and foetus was perhaps the greatest challenge of all.

As previously said, for the clinical team involved, we acknowledge this was a particularly stressful episode, not only in consideration of the direct responsibility for the patient and her foetus’ life, but also because of the challenges in finding a balance between medical paternalism and decision-making liberalism. Although the principle of respect for personal autonomy is well established in our team’s clinical practice, in such a complex decision making situation, the wider involvement of our MDT was paramount: it assisted with patient understanding of advanced medical information and provided further psychological support. Therefore, precisely for these reasons, the great effort provided by the MDT in order to combine clinical aspects with compassionate support in providing holistic care cannot go unnoticed.

In conclusion, we present a rare case with deep reflection on ethical dilemmas experienced by the MDT and the mental well being of the patient during the COVID-19 pandemic. The challenges represented by the diagnosis of neoplasia, especially in a young woman during her first pregnancy, can be a destructive event from multiple perspectives; and for this reason, it is fundamental that there is involvement of suitable clinicians to manage the case both from medical and psychological aspects. They warrant a full complement of multidisciplinary team specialists’ knowledge and expertise. We acknowledge a paucity of scientific literature available on this topic; and so, in addition to being a case of interest to the medical community, we hope that this manuscript will add to the growing evidence base on the appropriate management of cervical cancer during pregnancy. We report our results in the context of clinical practice to highlight such unusual circumstances where guidelines do not exist, to aid clinicians further in their future provision of treatment. We have provided an opportunity to focus the attention of the reader not only on improved strategies for management of cervical cancer patients in pregnancy from a clinical point of view, but also from an ethical and emotional stance. We promote our recommendations for healthcare professionals to always consider the holistic dimension of the patient’s life in their practice and to remember to treat the patient and not only the disease.

## 4. Informed Consent

During this patients treatment the surgical team sought and obtained formal permission from the patient to report the information as above. Ethic’s committee approval was not deemed necessary.

## Data Availability

Not applicable.
